# Correcting for respiratory motion in liver PET/MRI: preliminary evaluation of the utility of bellows and navigated hepatobiliary phase imaging

**DOI:** 10.1186/s40658-015-0125-0

**Published:** 2015-09-18

**Authors:** Thomas A. Hope, Emily F. Verdin, Emily K. Bergsland, Michael A. Ohliger, Carlos U. Corvera, Eric K. Nakakura

**Affiliations:** Department of Radiology and Biomedical Imaging, University of California, San Francisco, San Francisco, CA USA; Department of Radiology, San Francisco VA Medical Center, San Francisco, CA USA; Division of Hematology/Oncology, Department of Medicine, University of California, San Francisco, San Francisco, CA USA; Department of Radiology, San Francisco General Hospital, San Francisco, CA USA; Division of Surgical Oncology, Department of Surgery, University of California, San Francisco, San Francisco, CA USA

## Abstract

**Background:**

The purpose of this study was to evaluate the utility of bellows-based respiratory compensation and navigated hepatobiliary phase imaging to correct for respiratory motion in the setting of dedicated liver PET/MRI.

**Methods:**

Institutional review board approval and informed consent were obtained. Six patients with metastatic neuroendocrine tumor were imaged using Ga-68 DOTA-TOC PET/MRI. Whole body imaging and a dedicated 15-min liver PET acquisition was performed, in addition to navigated and breath-held hepatobiliary phase (HBP) MRI. Liver PET data was reconstructed three ways: the entire data set (liver PET), gated using respiratory bellows (RC-liver PET), and a non-gated data set reconstructed using the same amount of data used in the RC-liver PET (shortened liver PET). Liver lesions were evaluated using SUV_max_, SUV_peak_, SUV_mean_, and Vol_isocontour_. Additionally, the displacement of each lesion between the RC-liver PET images and the navigated and breath-held HBP images was calculated.

**Results:**

Respiratory compensation resulted in a 43 % increase in SUVs compared to ungated data (liver vs RC-liver PET SUV_max_ 26.0 vs 37.3, *p* < 0.001) and a 25 % increase compared to a non-gated reconstruction using the same amount of data (RC-liver vs shortened liver PET SUV_max_ 26.0 vs 32.6, *p* < 0.001). Lesion displacement was minimized using navigated HBP MRI (1.3 ± 1.0 mm) compared to breath-held HBP MRI (23.3 ± 1.0 mm).

**Conclusions:**

Respiratory bellows can provide accurate respiratory compensation when imaging liver lesions using PET/MRI, and results in increased SUVs due to a combination of increased image noise and reduced respiratory blurring. Additionally, navigated HBP MRI accurately aligns with respiratory compensated PET data.

## Background

The introduction of simultaneous PET/MRI promises to combine the soft tissue resolution associated with MRI and the high sensitivity and specificity of PET imaging. One of the difficult aspects of simultaneous imaging is how to appropriately leverage prolonged single bed position MR imaging into a whole body PET protocol [1–3]. This issue is fairly straightforward for brain and pelvis applications as there is minimal motion associated with the imaging and boils down to MR sequence selection. For chest and abdominal applications, such as liver imaging, respiratory motion can create issues for both MRI and PET imaging [4, 5].

**Fig. 1:**
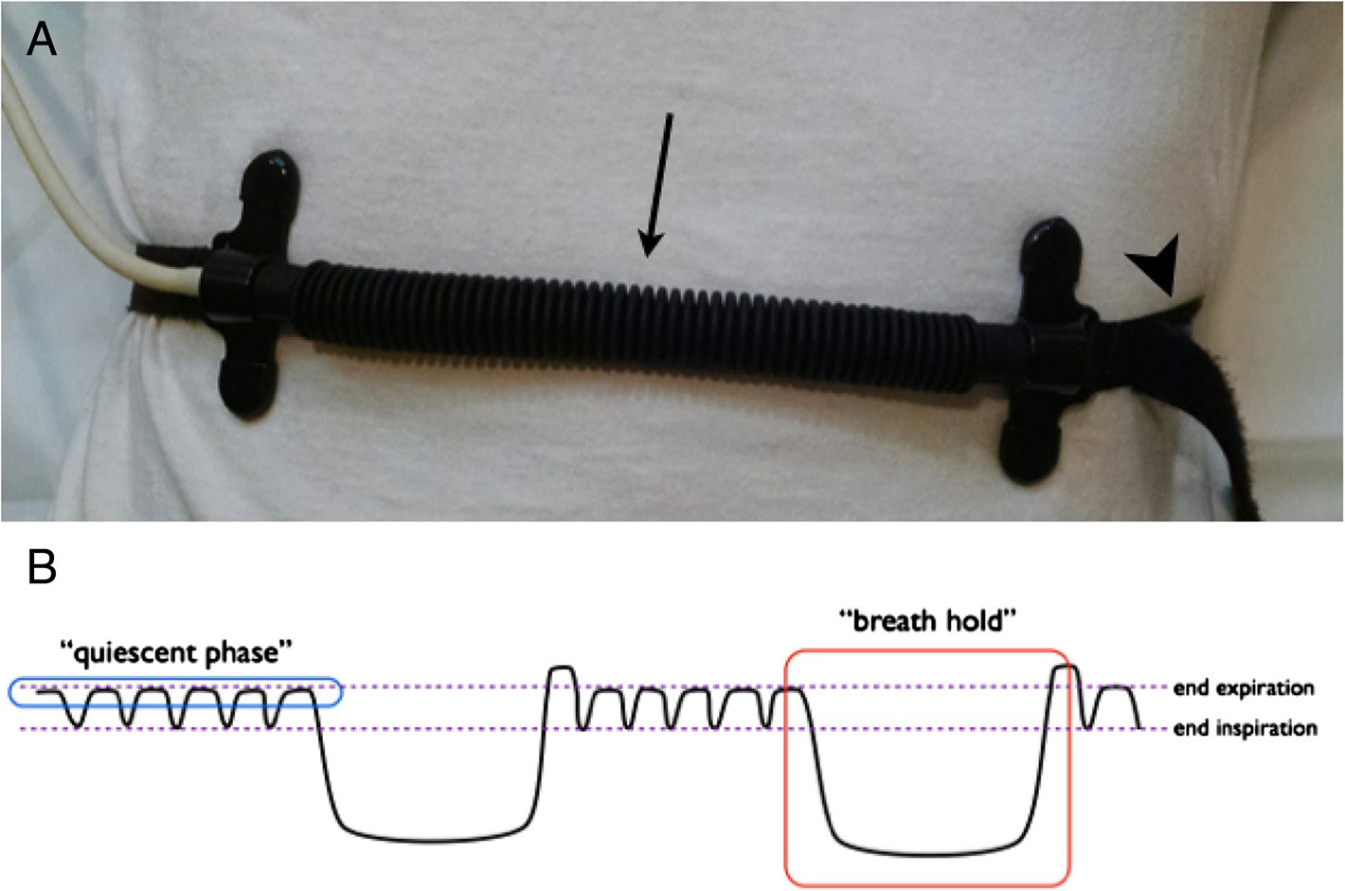
Using bellows for respiratory motion. Respiratory bellows are placed over the diaphragm or abdomen where excursion is greatest from respiratory motion, and are attached with a Velcro strap (**a**, *arrowhead*). A corrugated pneumatic tube (**a**, *arrow*) expands and contracts with each respiration, and pressure changes from within the tube are transmitted to a transducer through rubber tubing. **b** demonstrates a representation of a bellows tracing. Breathing at rest has decreased excursion of the diaphragm that is periodic. During breath-hold imaging, patients take a breath deeper and hold their diaphragm location for an extended period of time (15–30 s), measured as decreased pressure within the tubing during inspiration (**b**, *breath hold*). The goal of respiratory gating is to collect data that is acquired during the quiescent phase, which is closest to end expiration (**b**, *quiescent phase*)

Unlike conventional PET/CT acquisitions, patients perform numerous breath-holds throughout dedicated liver MR imaging markedly displacing the anatomy throughout the acquisition (Fig. 1). This can result in motion artifacts and errors in quantification. Additionally, localization of PET data to specific lesions seen on MRI can be difficult due to the displacement. MR sequences are often acquired during inspiratory breath-holds, while PET data is predominantly acquired during end expiration.

Therefore, there is a need to develop respiratory compensation techniques to remove respiratory artifact from the PET data, as well as techniques to acquire MRI images that accurately localize with the PET data. In this study, we imaged six patients with metastatic neuroendocrine tumor to the liver using Ga-68 DOTA-TOC PET/MRI. We evaluated the use of respiratory bellows-compensated PET and navigated hepatobiliary phase imaging in order to address the issues with respiratory motion in liver imaging.

## Methods

This study was approved by the local institutional review board, the UCSF Committee for Human Research and informed consent was obtained on all patients. Six patients were imaged between October 2014 and April 2015 with an average age of 65.5 years (three men and three women). Three of the patients were previously reported [6].

### Imaging protocol

Patients were imaged on a 3.0T GE Signa PET/MRI (GE Healthcare, Waukesha, WI). Each patient was injected with 5.0 ± 0.6 mCi of Ga-68 DOTA-TOC. PET/MRI imaging began an average of 117 ± 7 min after injection. The acquisition was delayed due to a preceding PET/CT each patient had; this delay results in a decreased amount of PET activity available for the PET/MRI acquisition. Each exam began with a six-bed position whole body PET/MRI with 2:15 s of acquisition time for PET at each bed position. During the whole body PET acquisition, a single breath-hold was performed for the T1-spoiled gradient echo acquisition. Subsequently, a liver specific bed position was acquired. During this bed position, precontrast (breath-held), dynamic postcontrast spoiled gradient echo imaging (breath-held), axial (breath-held) and coronal (free-breathing) single shot fast spin echo T2-weighted imaging, axial diffusion weighted imaging (free-breathing), and finally an axial navigated (free-breathing) and a breath-held hepatobiliary phase (HBP) spoiled gradient echo imaging were performed with the following parameters: slice thickness = 4 mm, flip angle = 35°, matrix size = 320 × 224, TE/TR = 2.0/5.5, NEX = 0.7. Patients were injected with 10 mL of gadoxetate disodium (Eovist, Bayer Healthcare), and HBP imaging was performed 10–20 min after injection when the hepatic parenchyma was enhanced due to hepatobiliary excretion. For breath-held imaging, patients were asked to take in a breath and hold their breath. Navigation was performed using a pencil navigator over the diaphragm, and MR data was acquired during end expiration [7]. In short, a pencil navigator acquires a linear excitation through the dome of the liver continuously throughout the acquisition and is used to track to location of the diaphragm. A high flip angle was used due to the increased intrinsic T1 contrast during the HBP [8].

### PET reconstruction

**Fig. 2:**
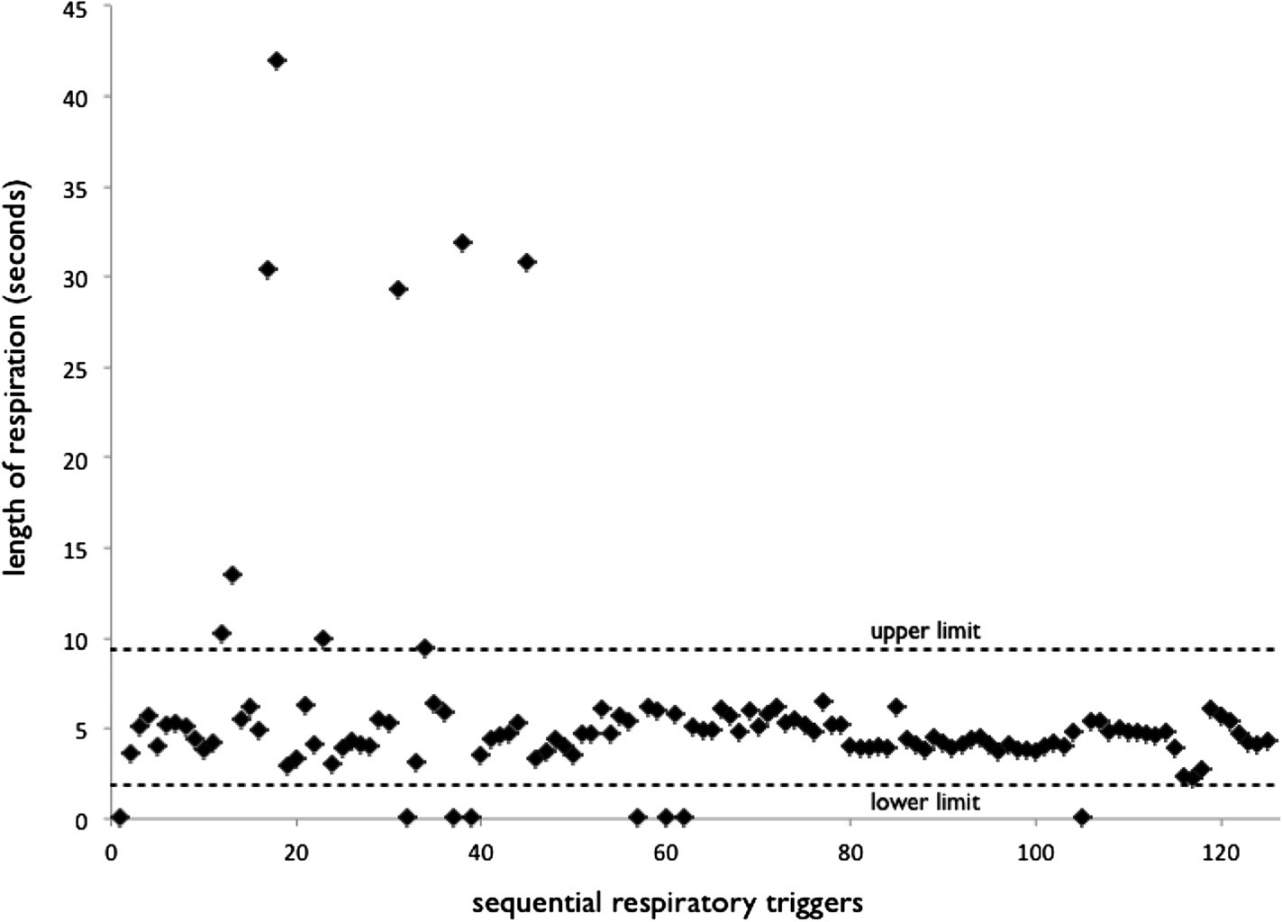
Example distribution of respiration length during a 15-min PET liver acquisition. The upper and lower limits for including PET data for the respiratory compensated reconstruction are noted by the *dashed lines*. Note the multiple long breath-holds during the early part of the scan, which correlate with the dynamic contrast enhancement and SSFSE breath-holds

All of the following PET data sets were reconstructed using a time-of-flight reconstruction with OSEM using two iterations and 28 subsets, and a matrix size of 256 × 256. The PET transaxial and z-axis field of view are 600 and 250 mm, resulting in a voxel size of 2.3 × 2.3 mm. Axial slices were reconstructed at 2.78 mm in thickness. Attenuation correction was performed using a two-echo Dixon fat-water separation algorithm for the body while the lung was segmented using a region growing algorithm, which is standard on the scanner [1]. Attenuation correction MR data was acquired during shallow breathing. Using respiratory triggers obtained from bellows, the following PET data reconstructions were obtained of the liver:

1. WB PET: non-gated PET from the whole body PET/MRI, a 2:15 s acquisition.
2. Liver PET: non-gated PET from the 15-min liver bed position. Dedicated liver PET imaging began an average of 31 ± 7 min after the beginning of the whole body acquisition.
3. RC-liver PET: respiratory compensated (Qstatic, GE Healthcare, Waukesha, WI) PET data from the 15-min liver bed position [9]. A respiratory bellows is a pressure sensitive band that surrounds a patient’s abdomen; as a patient breathes, the band expands and contracts to create respiratory waveforms (Fig. 1) [10]. Using the waveform from the bellows, respiratory triggers are created to denote the beginning of each inspiration. Fifty percent of the respiratory period from accepted breath-holds was included in the final reconstruction in order to use data only during end expiration when diaphragm motion is minimized. The 50 % window began at a 30 % delay from the bellows trigger so encompassed data between 30 and 80 % of the respiratory cycle relative to the bellows trigger. Any respiration that is longer than 10 s or less than 2 s in duration was excluded. On average, there were 91 accepted triggers and 25 rejected triggers from the total PET acquisition. On average 3.3 ± 1.3 min of PET data was used to reconstruct the RC-liver PET data sets.
4. Shortened liver PET: because changes in SUV can be due to both changes in noise and respiratory compensation, we created a “shorted” PET reconstruction that did not incorporate respiratory compensation, but used the same amount of PET data as the RC-liver PET reconstruction. For this reason, shortened non-gated PET data from the 15-min liver bed was reconstructed utilizing the same amount of PET data used to make the RC-liver PET data set. Respiratory trigger data was evaluated to only include PET data acquired during regular respirations in order to exclude long breath-holds in this data set (Fig. 2).

### Qualitative evaluation of motion artifact

The four PET reconstructions were graded qualitatively for the presence of motion artifact. Qualitative characterization was performed on coronal reformats. Motion was graded on a linear 1–5 scale (1: no motion artifact, 2: mild blurring without ghosting, 3: significant blurring without ghosting, 4: blurring and minimal ghosting, 5: marked blurring and ghosting lesions).

### Effect of respiratory gating on SUV measurements

Hepatic lesions between 1 and 5 cm in diameter that are distinct from adjacent lesions were included for analysis. Additionally, lesions that were not able to be segmented using a threshold due to low uptake compared to the background liver or difficulty segmenting from adjacent lesions were excluded. For each lesion, the SUV_max_, SUV_mean_, and SUV_peak_ were calculated. SUV_peak_ is measured as the average value in a 1-cm^3^ sphere centered around the maximum voxel within an ROI. SUV_mean_ and Vol_isocontour_ were calculated using a threshold-based technique with an isocontour line defined by 42 % of the maximum activity within the ROI. All measurements were performed using an Advantage Workstation 5.0 (GE Healthcare, Waukesha WI).

### Lesion excursion evaluation

Hepatic lesions between 1 and 5 cm in diameter that are distinct from adjacent lesions were included for analysis. The center of each lesion on coronal MIP PET images was determined using Qstatic reconstruction, and the z-axis location was recorded. Similarly, the z-axis location of the center of each lesion was also recorded on coronal reconstructions of breath-held and navigated hepatobiliary imaging. The difference in location between the MR and PET acquisitions was then calculated.

### Noise evaluation

Noise was evaluated as previously described for liver parenchyma. A region of interest (ROI) was placed manually over liver parenchyma in the left and right hepatic lobes being careful not to include any focal lesions. Additionally, an ROI was placed anterior the patient in a region of uniform background activity. Noise is defined as the standard deviation of signal intensity of the liver divided by the average signal intensity of the liver within each ROI [11].

### Statistical analysis

All scale variables are presented as means and standard deviations. Comparison of means was performed using a paired Student’s *t* test. The Wilcoxon signed-rank test was used to compare qualitative scoring of motion artifact. A *p* value less than 0.05 was considered significant. All analyses were performed using R [12].

## Results

**Fig. 3:**
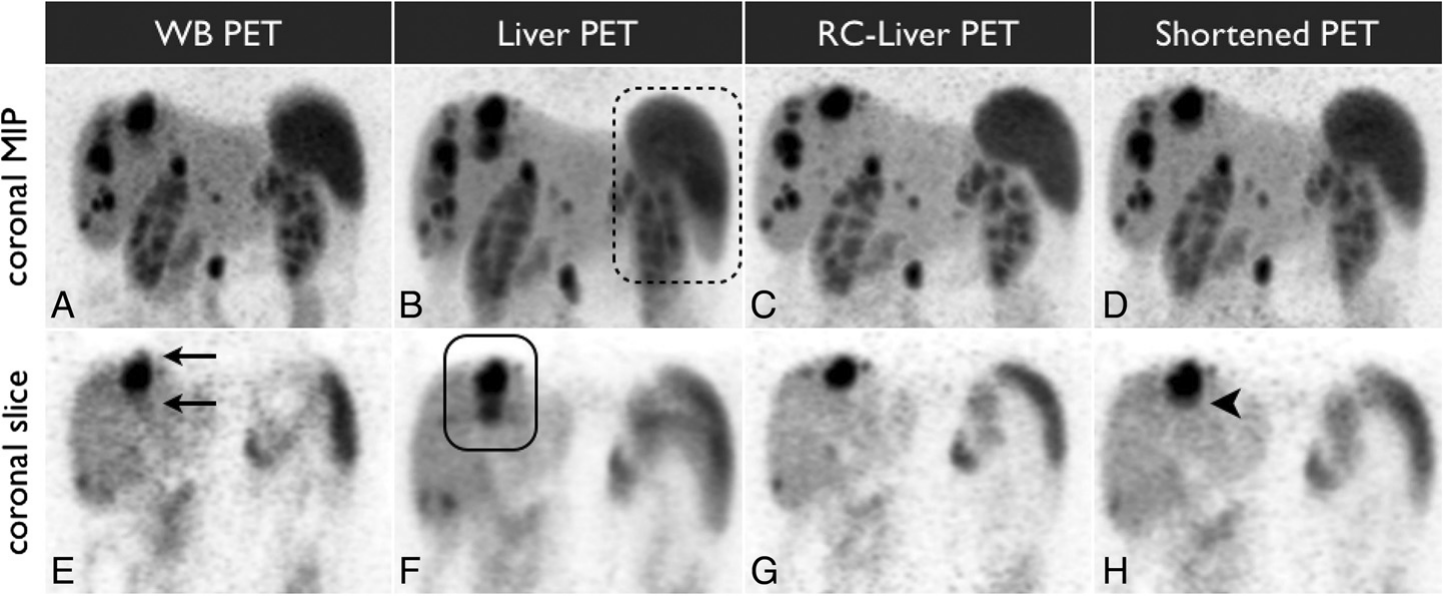
Example improvement in lesion delineation using a respiratory compensated reconstruction. Four PET data sets were reconstructed. The first was the PET data acquired during the whole body acquisition (WB PET, **a** and **e**), which demonstrates mild ghosting and blurring (**e**, *black arrows*). The non-respiratory compensated full data set from the liver bed position (liver PET, **b** and **f**) demonstrates respiratory ghosting of multiple avid liver lesions (*solid black box*) as well as the spleen (*dotted black box*). Reconstructions using respiratory compensation (RC-liver PET, **c** and **g**) remove the respiratory ghosting of both the liver lesions and spleen. Reconstructing the liver bed position using non-respiratory compensated PET data using the same amount of time as the RC-liver PET acquisition (shortened PET, **d** and **h**) demonstrates blurring associated with respirations (**h**, *black arrowhead*)

All imaged patients had DOTA-TOC avid hepatic lesions. Evaluation of breath-hold length demonstrated that there were multiple long breath-holds throughout the liver acquisition (Fig. 2), which correlates with breath-hold MRI sequences such as the dynamic contrast enhancement and single-shot fast spin-echo sequences (SSFSE). Qualitatively, the hepatic lesions demonstrated respiratory blurring and ghosting that was removed when applying bellows-based respiratory compensation (Fig. 3). Qualitative motion artifact was significantly less in the liver PET compared to the three other PET reconstructions (Table 1, *p* values ranging from <0.001 to 0.03).

**Table 1:** Average SUV_max_, SUV_mean_, SUV_peak_, Vol_isocontour_, and artifact score for each of the four PET reconstructions

	WB PET	Liver PET	RC-liver PET	Shortened PET
SUV_max_	33.0 ± 9.8*	26.0 ± 8.8*	37.3 ± 13.3	32.6 ± 10.5*
SUV_mean_	20.2 ± 6.3*	15.8 ± 5.2*	23.4 ± 8.7	20.4 ± 6.5*
SUV_peak_	23.5 ± 8.5*	20.0 ± 7.5*	27.6 ± 12.0	24.5 ± 9.4*
Vol_isocontour_ (cm^3^)	4.3 ± 4.5	5.4 ± 4.5*	3.7 ± 3.6	4.4 ± 4.3*
Artifact score	3.2 ± 0.8*	4.5 ± 0.5*	1.2 ± 0.4	2.7 ± 1.2*

*p value <0.05 compared to RC-liver PET

### Effect of respiratory gating on SUV measurements

**Fig. 4:**
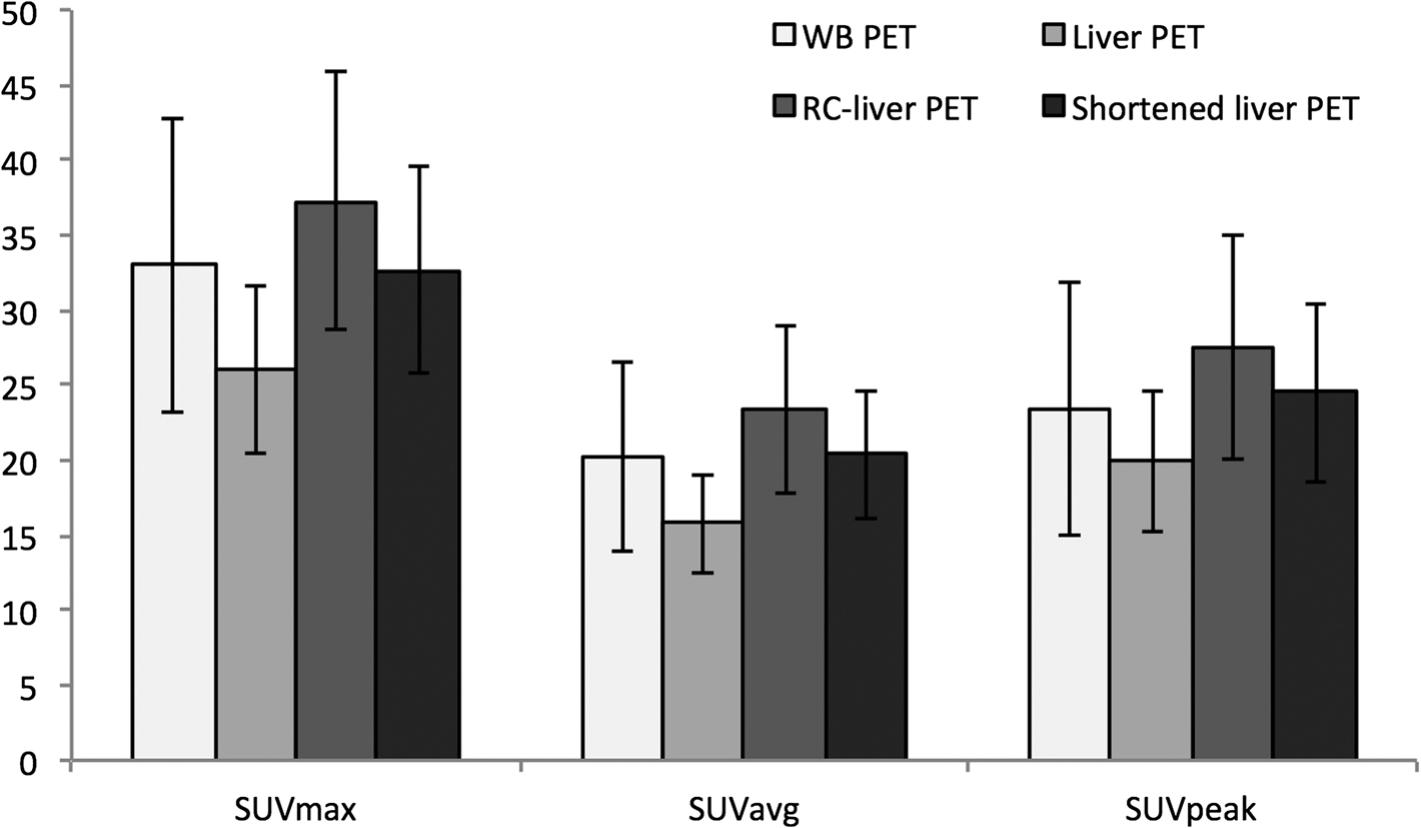
Average SUVs across measured lesions using the four PET data sets. The 15-min liver bed acquisition (liver PET) resulted in lower SUVs compared to the respiratory compensated reconstruction (RC-liver PET). Although simply reconstructing the liver PET data using the identical amount of counts used in the RC-liver PET reconstruction (shortened liver PET) also results in increased SUVs as well, although to a lesser extent than the RC-liver PET reconstruction. WB PET refers to the PET acquired of the liver during the whole body acquisition

Twelve individual lesions were included for analysis (Fig. 4, Table 1). RC-liver PET had a SUV_max_ and SUV_peak_ greater than the liver PET (43 and 38 % greater, both *p* values <0.001) and the shortened liver PET (14 and 13 % greater, *p* < 0.001 and *p* = 0.008). The shortened liver PET had a SUV_max_ and SUV_peak_ greater than the liver PET (25 and 23 % greater, both *p* values <0.001).

Respiratory compensation (RC-liver PET) resulted in a significant decrease in Vol_isocontour_ compared to the liver PET (3.7 ± 3.6 cm^3^ vs 5.4 ± 4.5, 31 % decrease; *p* = 0.001). There was significantly larger Vol_isocontour_ with the liver PET compared to the WB PET likely due to the numerous breath-holds performed during the dedicated liver imaging (25 % greater, *p* = 0.009).

### Lesion excursion

**Fig. 5:**
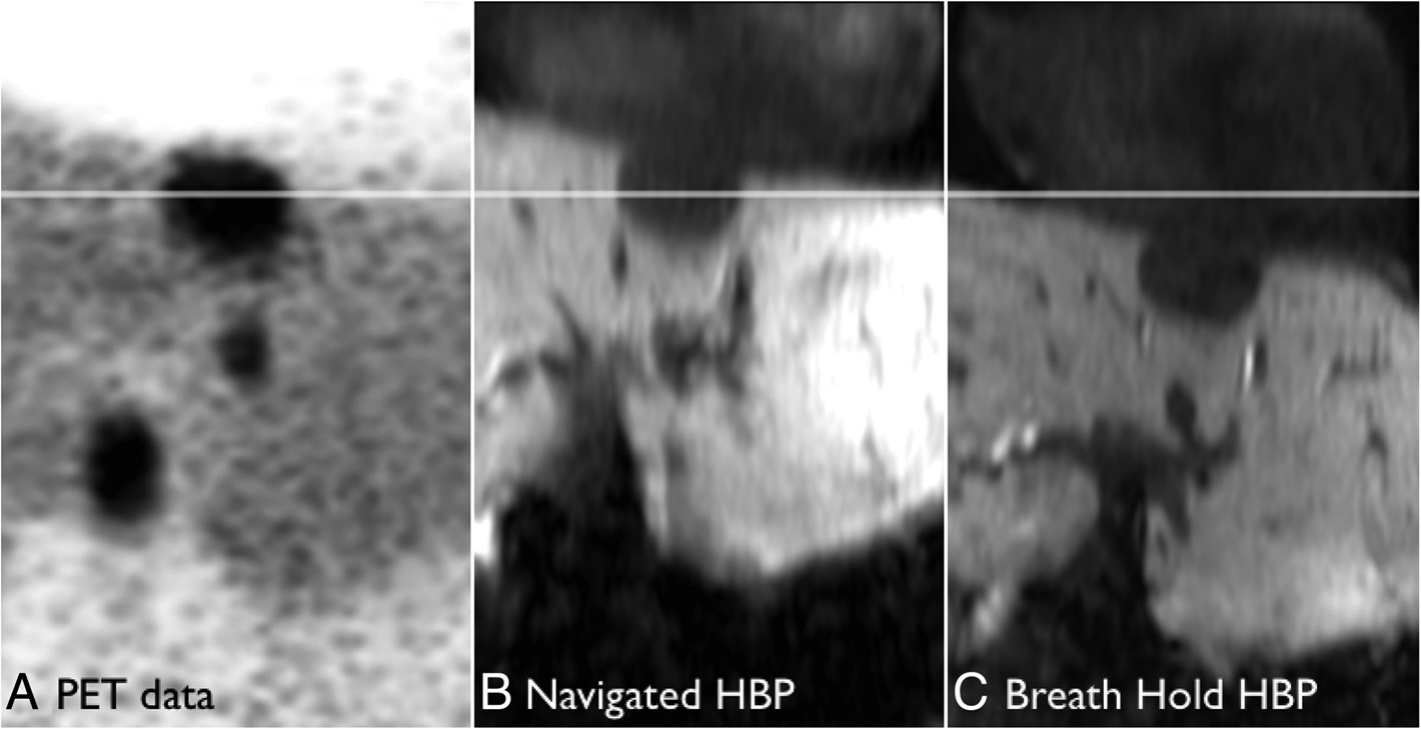
Example case demonstrating accurate localization of MR and PET data using navigated MRI imaging. Respiratory compensated (RC-liver) PET data (**a**) demonstrates a lesion in segment 2/4A of the liver, which corresponds with a hepatobiliary phase (HBP) hypointense lesion on MR (**b** and **c**). Using a navigated acquisition, the lesion is aligned with the PET data (**a** and **b**), as compared to a breath-held acquisition where the lesion is displaced inferiorly with respect to the PET acquisition (**a** and **c**)

**Fig. 6:**
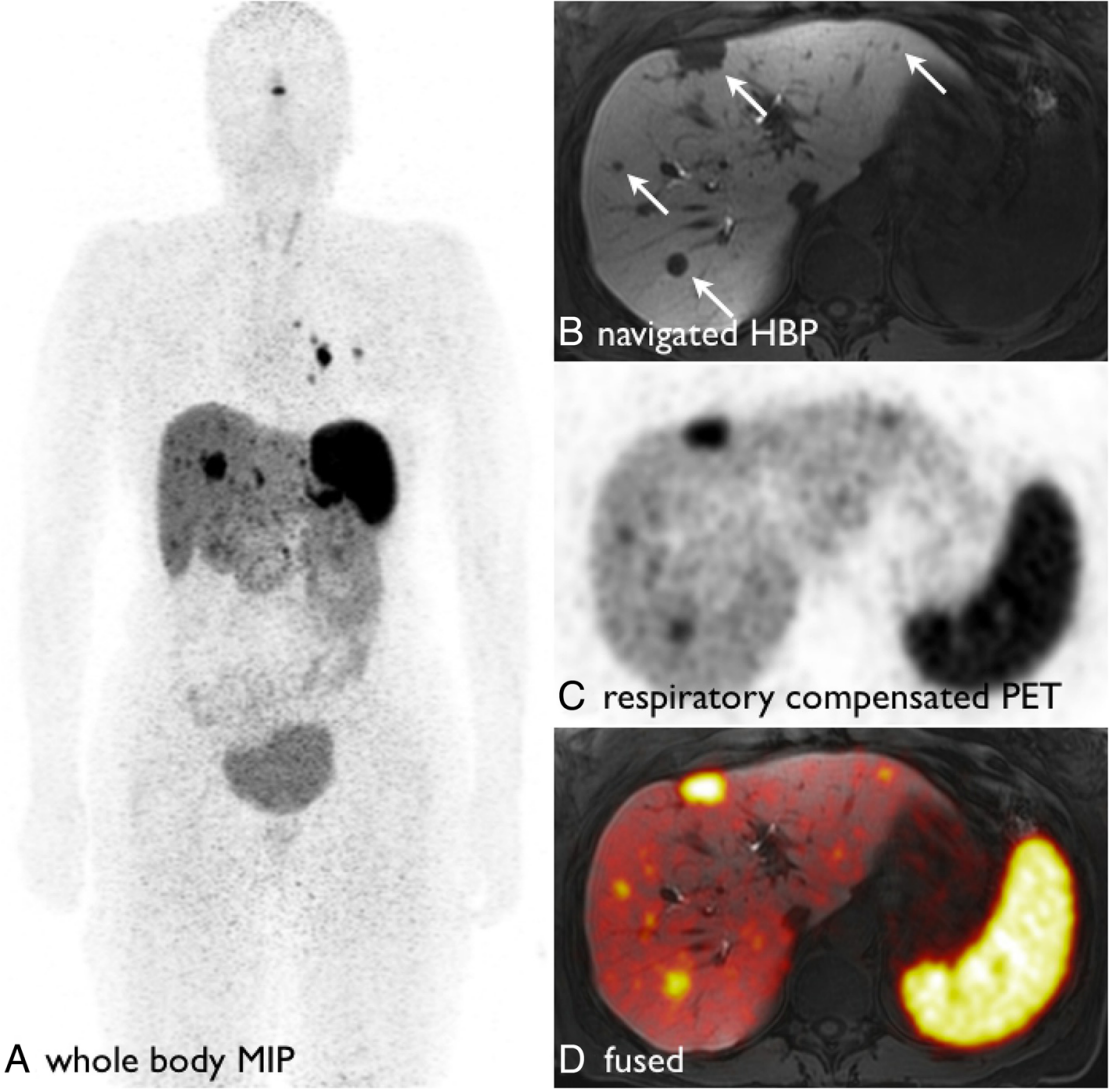
A 57-year-old female with metastatic neuroendocrine tumor to the liver, the mediastinum, and lung (**a**). Hepatobiliary phase imaging of the liver demonstrates multiple hypointensities (**b**, *arrows*) that correlate with DOTA-TOC uptake on respiratory compensated PET imaging (**c**). By combining the navigated HBP imaging and respiratory compensated PET data, accurate fusion between PET and MRI can be performed (**d**)

Sixteen individual lesions were included in the lesion excursion evaluation. Navigated HBP MRI resulted in accurate registration to the RC-liver PET data, compared to breath-held MRI where there was noted misregistration (Fig. 5). The average distance between the center of the lesion on RC-liver PET and on breath-held MRI was 23.3 ± 8.1 mm (*p* < 0.001), while the average distance between the center of the lesions on RC-liver PET and on navigated MRI was 1.3 ± 1.0 mm (*p* = 0.3). Fusion using navigated HBP imaging and respiratory compensated PET data resulted in accurate fusion (Fig. 6).

### Noise evaluation

The average noise measurements in the liver for the WB PET, liver PET, RC-liver PET, and shortened liver PET were 0.23, 0.13, 0.23, and 0.21, respectively. The noise in the liver PET was significantly lower than in the RC-liver PET and shortened liver PET (*p* = 0.016 and 0.019). There was no significant difference in noise between the RC-liver PET and shortened liver PET (*p* = 0.22).

## Discussion

We have demonstrated that the incorporation of respiratory compensation into PET reconstruction can aid in removing respiratory artifact in PET/MRI. Using bellows is a convenient way to incorporate respiratory gating without affecting MRI imaging that is already included in clinically available scanners. Of note, the measured SUVs were higher using respiratory compensation (RC-liver PET) due to increased image noise and removal of motion artifact compared to the full acquisition (liver PET), and when corrected for scan time (shortened liver PET), respiratory compensation resulted in a slight increase in SUVs. Additionally, using a navigated hepatobiliary phase resulted in improved alignment of PET and MRI data allowing for accurate fusion.

There are two reasons that SUV values would be different when incorporating respiratory compensation. First, respiratory compensation decreases the amount of PET data used in reconstruction resulting in more noise in the data set. Previous work using FDG has demonstrated that SUV_max_ calculations are sensitive to noise and values decrease with longer scan times as image noise decreases [13]. Separate from the issue of noise, the removal of respiratory blurring will increase measured SUVs [14, 15]. In order to separate these two effects, we reconstructed a non-respiratory compensated data set (shortened liver PET) that used the identical amount of data as in the respiratory compensated reconstruction (RC-liver PET) in order to create datasets with the same amount of noise. We demonstrated that the effect of increased noise and removal of respiratory blurring both resulted in increased SUVs as expected. Previous work has also shown that respiratory gating in the setting of PET/MRI results in increased SUVs but did not take into account changes in image noise [16].

Similar approaches to performing respiratory compensation have been used in abdominal PET/CT using sensor belts around the abdomen [17, 18]. Other techniques have used a positioning monitoring system that involves an infrared camera, although this may not be translatable to the PET/MRI setting due to the narrow bore size, anterior coils, and the magnetic field [19]. It is also possible to use MRI navigators to provide gating information in order to reconstruct respiratory gated PET data [16, 20, 21]. This approach is limited as it requires MRI data to be continuously acquired throughout the PET acquisition, limiting what MR data you can acquire. Other approaches use MR data to create deformation maps that allow one to register PET data acquired at different time points in the respiratory cycle to create a respiratory gated data set without removing motion corrupted data [22]. Finally, one can use the PET data itself to perform motion correction although these approaches are limited due to noise [23, 24]. One recent interesting approach is to use a training dataset to inform how respiratory motion can be gated using list mode PET data itself, although this requires a short acquisition after the completion of the study [5]. Overall, we believe the use of bellows-based respiratory gating provides a simple robust way to gate PET/MRI data without affecting MR imaging.

We report the first use of diaphragm-navigated HBP imaging for PET/MRI and demonstrated that gated MR acquisition during end expiration results in an MR image that accurately fuses with respiratory compensated PET data. Other approaches to address this issue could be to perform a radial free-breathing acquisition as it results in an image that is an average throughout free-breathing similar to PET data, although this has not been evaluated in the setting of PET/MRI [25].

One important point is that image noise greatly influences SUVs. Typically PET/CT is done with a standard amount of time at each bed position. With PET/MRI, due to the need to perform numerous MRI sequences [26], one may decide to increase the amount of time acquiring data at a single bed position. Although the increased frame time can increase lesion detection and improve image quality, the differences in frame time can result in nearly 30 % differences in SUVs. Therefore, when evaluating for changes in SUV, it is critical to choose PET data sets with similar frame times.

There are a number of limitations associated with this study. First is the limited number of patients and lesions imaged, which will require further work to validate the findings in this study. Second, the same attenuation correction map was used for all PET data reconstructions, and so differences in SUV values may be due to misregistration between PET and MRAC caused by respiratory motion [27]. Third, the increased delay time for PET/MRI compared to routine clinical imaging time points of 55–70 min results in decreased activity resulting in more noise than may be present at shorter imaging delays.

## Conclusions

In conclusion, we have demonstrated the utility of respiratory bellows for providing respiratory compensation when imaging liver lesions using PET/MRI using existing software available on clinical PET/MR systems. Respiratory compensation results in increased SUVs both due to increased image noise and reduced respiratory blurring. Finally, navigated HBP MRI provides high quality images that accurate align with respiratory compensated PET data.

## Ethical approval

All procedures performed were in accordance with the ethical standards of the local institutional research committee.

## Informed consent

Informed consent was obtained from all individual participants included in the study.
